# Assessment of Quality of Life of Women with Breast Cancer

**DOI:** 10.5539/gjhs.v8n9p1

**Published:** 2015-10-16

**Authors:** Zivana Gavric, Zivana Vukovic-Kostic

**Affiliations:** 1 The Public Health Institute Republic of Srpska, Jovan Ducic 1, Banja Luka, Bosnia nad Herzegovina; 2 Faculty of Medicine University Banja Luka, Banja Luka, Bosnia and Herzegovina; 3 Health Insurance Fund Republic of Srpska, Bosnia and Herzegovina

**Keywords:** women, quality of life, breast cancer

## Abstract

**Background:**

Breast cancer is the most common type of cancer among women in 145 countries worldwide, and the success of healthcare for women with this disease is measured with the quality of life of survivors. The aim of this study was to examine how the breast cancer affects the quality of life and in what dimension of health quality of life is the least accomplished.

**Method:**

A pilot research had been performed in the period from June 10 to August 15 2011, on 100 women from Association of women with breast cancer “Iskra” in Banja Luka, aged 20-75. The survey research was based on the EORTC QLQ-C30 version 3.0 and questionnaire for assessment of quality of life of those suffering from breast cancer QLQ-BR23 with 53 questions in total.

**Results:**

The average age of women in research was 51.8 years (±11.23). Statistically important differences (χ^2^_4_=221.941; p<0.01) are higher mean values of the score for the functional scale, (66.32±17.82) cognitive functions (63.50±28.00) in relation to functional role (46.83±20.88), social (37.00±27.58) and emotional (36.58 ±25.15) functioning. Mean values of the score for the symptoms scale were statistically higher for symptoms such as fatigue, insomnia and pain in relation to other symptoms. Mean values of the score for body image scale are statistically higher in relation to mean values of the score of sexual functions and enjoyment scale, and the scale for grading the future perspectives.

**Conclusion:**

Breast cancer affects all the domains of the quality of life, and in our population it is the most prominent in domains of emotional and social functions, as well as role functions. Symptoms of fatigue, insomnia and pain have the most importance influence on these domains.

## 1. Introduction

According to the newest Globocan 2008 database, issued by the International Agency for Research on Cancer (IARC), 1.384.000 women get breast cancer per year. Of estimated 1, 38 million of new cases or 23% of all cancers, breast cancer takes the second place in overall cancer occurrence or 10, 9% of all cancers. It is also the most common cancer of women in 145 countries of the world, and the incidence rates vary from 19, 3 in 100.000 women in East Africa, to 89, 7 in 100.000 women in Western Europe, thus being extremely high (more than 80 in 100.000) in developed parts of the world (except in Japan) and low (less than 40 in 100.000) in most countries of regions in development (WHO, 2010).

Cancer treatment makes ten year survival relatively easy to achieve, and the psychological, sexual and physical dysfunctions caused by diagnostics and treatment of cancer affect the women’s quality of life. There is a relatively wide array of treatments with similar response to therapy and survival rates, but with enormous differences in effects on physical and emotional wellbeing of a woman (Victorson, Cella, Wagner, Kramer & Smith, 2007).

Survival is stated as the most relative therapeutically outcome for patients suffering from breast cancer, and little attention is given to physical limitations and other health problems that have effect on this, especially with elderly women suffering from breast cancer. Although these problems are affiliated with poor tolerance to treatment, it is unclear whether these limitations have an effect on the risk from death by death cancer or other competing causes (Worden & Weisman, 1997).

Cancer treatment has a goal to offer the best possible chances for recovery. However, even the best treatments have side effects, some of which include: fatigue, hair loss, short-term limited hand movements. Even though every person experiences these side-effects in a different way, changes in the physical appearance often lead to changes in body image, which brings stress and anxiety. Breast cancer can affect the patient psychologically, as well as her organs, and it can also manifest through post mastectomy depression, or increased anxiety, the feeling of humiliation with occasional suicidal tendencies (Goldwyn, 1987).

The quality of life related to health (Health Related Quality of Life-HRQoL) resulted as a consequence of understanding that health is an important, if not the most important condition of good quality of life (Paterson, 2010).

Torrance considers that, when it comes to assessment of quality of life with implemented therapy procedures after the diagnosis, two different factors should be taken into account: the length of patient’s life and the quality of patient’s life (Keszei, Novak, & Streiner, 2010).

In modern day worldwide literature, authors give more or less significance to different endogenous or exogenous factors which influence the quality of life, so there are different definitions of the quality of life. This is also the reason why it is hard to define a unique concept measurable in a way that it provides comparability. Quality of life is important for every human being, but this importance grows with the occurrence of disease, addiction and close end to life (Monteiro-Grillo, Marques-Vidal, & Jorge, 2005).

Though the concept of quality of life is often used, it is hard to define it. It depends on the subjective understanding of health and disease, it is multi-dimensional, and in accordance with the definition given by the World Health Organization (WHO) it consists of: physical, mental and social health, cognitive and sexual functions, working ability and life long pleasure (WHOQOL Group, 1993).

The physical domain refers to the subjective assessment of health condition and body functions (e.g. fatigue, incontinence, lymphedema), whilst emotional component covers psychological functions, including positive and negative mood indicators (e.g. anxiety, symptoms of depression…) and limitations caused by emotional problems. Sexual quality of life is generally referred to the perception of sexual functions, changes in sexual desires, and changes related to body image. The social domain often includes the affect of disease on the individual itself, the social role and perception of usefulness of social support. These domains are interrelated (Stanton, Revenson, & Tennen, 2007; Coleman, Quaresma, & Berrino, 2008).

In today’s age of increasingly competitive market of health, the problem represents measuring the quality of protection for certain diseases, as well as quality of life (QoL), and this has become a topic of great interest and controversy among the healthcare providers, as well as patients (Ganz et al., 2004).

The aim of this study was to examine how the breast cancer affects the quality of life and in which dimension of health quality is this least accomplished.

## 2. Method

The research had been performed in the period from June 10 to August 15 2011, among 100 women treated from breast cancer, from Association “Iskra” in Banja Luka.

Association “Iskra” from Banja Luka is an organization whose aim is to provide professional psychosocial support to women suffering from breast cancer and persons who survived breast cancer, by creating educational programs that deal with early detection of this disease.

All participants of the survey received a questionnaire that explains how to fulfill it, as well as a form where they signed consent on anonymity and they were given an option to refuse participation in the survey at any time, without given explanation.

Research was based on a questionnaire of the European Organization for Research and Treatment of Cancer (EORTC) –quality of life of patients with cancer QLQ-C30 (The Core Quality of Life Questionnaire of The European Organization for Research and Treatment of Cancer) (version 3.0) with 30 questions (Aaronson et al., 1993). The questionnaire consists of five functional scales: Physical functioning; Role functioning; Emotional functioning Cognitive and Social functioning; and three scales of symptoms: pain, nausea/vomiting, Dyspnoea, Insomnia, Appetite loss, Constipation and Diarrhoea.

According to EORTC QLQ-C30 Scoring Manual–” all scales and single-item measures are ranged score of 0-100. Higher scores in the rankings are the result of presenting a higher level of response. Such a high score with a functional scale represents a high/healthy level of functioning, a high score for the global health status/QOL represents a high quality of life-QOL, as well as a high score for a symptom scale/item that represents a high level of symptoms/problems (Fayers et al., 2001). A higher score represents a higher (“better”) level of functioning, or higher (“worse”) level of symptoms (De Haes, Von Knipperg, & Neijt, 1990). Criteria for inclusion in the study were: persons aged from 20-75 years, members of associations and voluntary participation.

Along with this questionnaire about quality of life another was used: a questionnaire about assessment of quality of life of those suffering from breast cancer QLQ-BR23 (Quality of Life Questionnaire Breast Cancer) with 23 questions (survey questionnaire added in attachment). Those 23 questions were divided into functional scales such as: body image, image of sexuality, future perspectives, symptoms of scale and one notion for assessment of systematic side-effects, symptoms of hand, chest and hair loss (Fayers et al., 2001).

Group for the quality of life of the European Organization for Research and Treatment of Cancer (EORTC Quality of Life Group), the questionnaire was translated into 55 languages, and their psychometric properties have been studied in different cultures (De Haes, Von Knipperg, & Neijt, 1990) and we are for research in the Republic of Srpska used QLQ-C30 Serbian.

The particles were graded according to Likert scale of 4 levels, ranging from 1 (not at all) to 4 (a lot), and a higher number of points denoted poor functioning, or a higher number of symptoms. The exception is the scale of general health/quality of life (QL2), which was graded with 7 points analogue, to the scale there higher number of points denoted higher satisfaction with general health condition and quality of life.

Analysis of the questionnaire after the survey was performed according to the manual Guidelines for determining the quality of life in EORTC Clinical Trials’ Group of the European Organization for Research and Treatment of Cancer EORTC quality of life QoQ (Young, De Haes, Curran, Fayers, & Brandberg, 1999). Depending on their nature, description of the parameters of interest was performed by means of descriptive statistics: frequency, percentage, means (average), median, standard deviation (CI) and range. Testing was performed with a significance level p <0.05 using Friedman test and Pearson χ^2^ test. Data were analyzed with statistically programs: SPSS for Windows software (SPSS13.0, Inc., Chicago, Illinois, USA) and Microsoft Excell (11. Corporation Microsoft, Redmond, WA, USA), representing the statistical parameters in the tables.

## 3. Results

In total 100 hundred of women from association “Iskra” suffering from breast cancer took part in the survey. The average age of the women was 51.8 years (±11.23), and more than ½ of women were aged 50 and over (Table 1).

**Table 1:** Distribution of sample by age

Age	women with breast cancer	

	n	%
≤30	4	4.0
31-49	43	43.0
50-59	32	32.0
60-69	13	13.0
≥70	8	8.0
***Total***	100	100.0

	Mean=51.8 (±11.23)* Min 28; Max 77	

* SD- Standard Deviation (±).

Statistically important differences are the higher medium values of the score for the functional scale, cognitive functions in relation to cognitive role, social and emotional functioning (Table 2).

**Table 2:** Score quality of life in women with breast cancer

*Scale*	*QLQ.C30. score Women with breast cancer

	Mean (SD)
	Median (Range)
hysical functioning	66.32 (±17.82)
	66.67 (26.67-93.33)
Role functioning	46.83 (±20.88)
	50.00 (0-83.33)
Emotional functioning	36.58 (±25.15)
	33.33 (0-83.33)
Cognitive functioning	63.50 (±28.00)
	66.67 (0-100)
Social functioning	37.00 (±27.58)
	33.33 (0-100)

* Friedman Test; χ^2^_4_=221.941; d f=4; p=0.00; SD- Standard Deviation (±).

Medium values of the score for the symptoms scale were statistically higher for symptoms such as fatigue, insomnia and pain in relation to symptoms of constipation, diarrhea, and loss of appetite, dyspnoea, nausea and vomiting (Table 3).

**Table 3:** Parameters score to QLQ.C30 symptom scales in women with breast cancer

*Scale*	*QLQ.C30. score for symptom scales

	Mean (SD)
	Median (Range)
Fatigue	53.22 (±20.79)
	55.56 (11-100)
Nausea and vomiting	33.83 (±25.45)
	33.33 (0-100)
Pain	50.33 (±24.04)
	50 (0-100)
Dyspnoea	25 (±26.54)
	33.33 (0-100)
Insomnia	53 (±21.76)
	66.67 (0-100)
Appetite loss	38 (±25.96)
	33.33 (0-100)
Constipation	9.33 (±17.78)
	0 (0-66.67)
Diarrhoea	38.33 (±29.73)
	33.33 (0-100)

* Friedman Test: χ^2^_4_=427.108; df=8; p=0.00; SD- Standard Deviation (±).

Values of the score of functional scale with women with disease show a statistically important difference in relation to medium values of the score of sexual functioning and pleasure scale, and scale for assessment of future perspectives (χ^2^_4_=147.079; d f=3; p<0.01) (Table 4).

**Table 4:** Parameters score for QLQ.BR 23 functioning scale in women with breast cancer

*Scale*	QOQ-BR23. Score for functioning scale

	Mean (SD)
	Median (Range)
Body image	51.03 (±32.1)
	50 (0-100)
Sexual functioning	18 (±18.9)
	16.7 (0-50)
Sexual enjoyment	10.33 (±17.53)
	0 (0-66.67)
Future perspective	14 (±26.46)
	0 (0-100)

Friedman Test: χ^2^_4_=147.079; d f=3; p=0.00; SD- Standard Deviation (±).

When it comes to values of the score of symptoms scale, the greatest medium values of the score are for symptoms of hair loss, with statistically important difference (χ^2^_4_=101.301; d f=3; p<0.01), in relation to other mean values of the scores for symptoms of hand, breast, and presence of therapy effects (Table 5).

**Table 5:** Parameters score for QLQ.BR 23 symptom scales in women with breast cancer

Scale	QLQ.BR 23. Score for symptom scales

	Mean (SD)
	Median (Range)
Systemic therapy side effects	43.12 (±19.72)
	42.86 (9.5-90.5)
Breast symptoms	32.57 (±20.24)
	33.33 (0-91.7)
Symptoms of hand	39.39 (±22.18)
	33.33 (0-100)
Upset by hair loss	64.31 (±32.03)
	66.67 (0-100)

* Friedman Test: χ^2^_4_=101.301; d f=3; p=0.00; SD- Standard Deviation (±).

## 4. Discussion

The questionnaire about the quality of life (QQL) is a module used by the European Organization for Research and Treatment of Cancer (EORTC). QLQ-C30 was tested on 170 Dutch, 168 Spanish, and 158 American women with breast cancer. The time of the survey for Dutch and Spanish women was before and during the radiotherapy treatment or chemotherapy (Sprangers et al., 1996). When it comes to American patients, the questionnaire was done during the registration to the clinic for breast, and three months after the first assessment. Cronbach alpha coefficient is, by rule, the lowest in Spain (ranging from 0.46-0.94) and the highest in the USA (ranging from 0.70-0.91). Based on the known groups of division, there are distinct differences among the patients with different stage of disease and with those who had previously had operations, with performance status and the way of treatment, as expected. The given results give full support to the clinical and intercultural importance of the questionnaire for quality of life of breast cancer patients QLQ-BR23 as an additional questionnaire for assessment of specific quality of life for patients with breast cancer (Sprangers et al., 1996).

In the study performed in Spain the relation between the pain thresholds in shoulder mobility, mood, perception of pain, muscular endurance, quality of life and fatigue among breast cancer survivors was surveyed. The research was performed in 59 women 6 months after the treatment. Amplitude of flexia of the shoulder was also done (McQuade test of flexor endurance) and the threshold of pain in C5-C6 joint, deltoid muscle, as well as an assessment of metacarpal and tibialis anterior muscle. The fatigue was higher with patients with higher depression (r = 0.45, P<0.05), and emotional scale indicated higher depression present with reduced body image and physical damage perception, as well as reduced shoulder mobility and multidimensional character of fatigue (Cantarero-Villanueva et al., 2011).

Social work was better among those with the breast cancer diagnosed in the period from 2-4 years. Pain, insomnia, loss of appetite and systematic effects of therapy as well as breast symptoms were more present in patients diagnosed with a year or less. Concerning the global health condition, relevant differences in relation to length of diagnosis was not noted. When speaking of symptoms, the highest values are for fatigue, financial difficulties, insomnia and pain, whilst for breast cancer the most specific symptoms are hair loss systematic therapy side-effects, symptoms related to hand and breast were higher with patients with shorter length of diagnosis (Gokgoz et al., 2011).

When speaking of the survey performed among the women suffering from breast cancer in Lebanon, the most frequent symptoms are the feelings of nervousness, sadness, lack of energy and pain (Abu-Saad Huijer & Abboud, 2012).

As a cross section to the survey performed in Yemen with 106 women with breast cancer who visited the infirmary in the national centre for oncology, an assessment of therapy of breast cancer in relation to social and demographic characteristics was done through this questionnaire. Years after the diagnosis, monthly family incomes and radiotherapy were statistically related with overall quality of life –QQL with patients with breast cancer (p=0.01, p=0.023, p= 0.039 respectively) (Al-Naggar, Nagi, Ali, & Almuasli, 2011).

In the AMBER (Alberta Moving Beyond Breast Cancer) Study in Alberta, Canada with 1500 newly diagnosed, incident, stage IIIc breast cancer survivors over a 5 year period is answered key questions related to physical activity and health-related fitness in breast cancer survivors and the independent and interactive associations of physical activity and health-related fitness with disease outcomes (e.g., recurrence, breast cancer-specific mortality, overall survival), treatment completion rates, symptoms and side effects (e.g., pain, lymphedema, fatigue, neuropathy), quality of life, and psychosocial functioning (e.g., anxiety, depression, self-esteem, happiness). Physical activity participation consistently decreases with advanced breast cancer and during treatments. This prospective cohort study is designed specifically to examine the role of physical activity and health-related fitness in breast cancer survivorship from the time of diagnosis and for the balance of life. The role of physical activity and health-related fitness is in facilitating treatment completion, alleviating treatment side effects, hastening recovery after treatments, improving long term quality of life, and reducing the risks of disease recurrence, other chronic diseases, and premature death (Courneya et al., 2012).

An online research in Australia, with 1.965 women suffering from breast cancer, containing 47 quantitative and qualitative notions, showed that reducing sexual functions and satisfaction contributed to a series of factors, including pain and fatigue, psychological unwillingness and body image, as well medically induced changes in the menopause such as dryness of the vagina, heat waves, and getting extra weight. Dominant problems identified in the qualitative analysis were emotional consequences, physical changes, feeling of unattractiveness, or lack of femininity, self-reconciliation regarding the changes, as well as influence on the partner or relations with him. This research indicated important changes in the sexual bliss after the diagnosis and treatment of breast cancer (Ussher, Perz, & Gilbert, 2012).

Surveying the occurrence of fatigue in women survivors from breast cancer in two large city areas on 1.956 women, in relation to general population and demographic and medical psychosocial component, showed that in average, the level of fatigue with survivors is somewhat more often in relation to general population of women same age, and that is was comparable and matching to women of the same age within the general population. Approximately one third of breast cancer survivors estimate a heavier fatigue, related to greatly higher levels of depression, pain and sleep disorders. Apart from that, fatigue bothers women in menopausal symptoms and probably more those who had received chemotherapy (with or without radiation) then in healthy women. In the multivariate analysis, depression and pain appeared as the strongest predictors of fatigue (Bower et al., 2000).

From overall 375 studies conducted in the USA, 20 quantitative and 2 qualitative studies were analyzed according to criteria included in the study. Quality of life of women with breast cancer QQL was monitored, comparing mental, physical, social and sexual scales of QQL among Latin American women and other racial/ethnic groups. Latin American women had a poorer mental, physical and social scale of quality of life (QQL) present, in relation to non-Latin American women. Of all the analyzed studies, the greatest difference among the four aspects was noted in the area of metal health, in which Latin American women displayed poorer scale in relation to non-white and Latin Negro women. The majority of quantitative research showed that Latin American women had lower quality of life (QQL), than non-Latin American women, for all the scales of measurement (6 studies) or it showed mixed findings in which Latin American women generally display poorer quality of life in the majority, but not all the scales of survey (12 studies) included in the study. Explanatory mechanisms were monitored, including socio-demographic characteristics, in relation to treatment, as well as culturally relevant factors. Implications for design survey, measurement and clinical work were also included, as well as implications for cancer survival. The data shows that the scales of the survivors score are in average lower in relation to non-white Latin American. Understanding the ethnic differences in the quality of life (QQL) among the cancer survivors can give information regarding interventions focused on improving the health condition for Latin American women (Yanez, Thompson, & Stanton, 2011). Research performed in 2009 with 192.000 American women living with breast cancer showed that exercise can help in the fight against the symptoms of breast cancer treatment, and fatigue was identified as potential obstacle in doing physical activities. Self-effectiveness can influence the cancer related fatigue and physical activities, and it improves the quality of life (QQL) subsequently. Women under the treatment fulfilled the surveys for demographic profile, scale of fatigue, questionnaire for assessment of physical activities and questionnaire for quality of life. The model was tested based on the responses of 73 women, and it showed 53% variations in results regarding quality of life (QQL), 28% variations regarding physical activity ad 31% variations regarding self-effectiveness. Even though fatigue is regarded as physical problem that demand physical intervention, this study provides new evidence that potential intervention for improving self-effectiveness can intermediate the effect of tumor that affects quality of life, and interventions on improving self-effectiveness can contribute to increasing physical activity and improving quality of life (QQL) in this population (Haas, 2011).

Medical condition or treatment had considerably more impact on family and social life of women with breast cancer with statistically significant difference compared to the control group women. Mean values of scores of emotional and social scales were significantly lower in ill women than in the control group of women. Research conducted regarding monitoring the quality of life of women with breast cancer have shown that in order to provide a better emotional and social functioning, it is necessary to ensure better support to the whole family, the environment and the community of women living with breast cancer (Gavric, 2015).

Conclusion: Breast cancer affects all the domains of quality of life, and in our population it is most prominent in the domain of emotional and social functioning, as well as functional role. Symptoms of fatigue, insomnia and pain have the most important influence on these components. In our sample breast cancer had the impact on functional scales as well; especially mean values of the score of body image scale, and the scale for symptoms with greatest mean values regarding hair loss.

When we speak about improving the quality of life of women with breast cancer, especially in the most impaired domains of health, it would be good to include a multidisciplinary team to work with affected women.

## References

[1] Aaronson N. K, Ahmedzai S, Bergman B, Bullinger M, Cull A, Duez N. J, Takeda F (1993). The European Organization for Research and Treatment of Cancer QLQ-C30: A quality-of-life instrument for use in international clinical trials in oncology. Journal of the National Cancer Institute.

[2] Abu-Saad Huijer H, Abboud S (2012). Health-related quality of life among breast cancer patients in Lebanon. Eur J Oncol Nurs.

[3] Al-Naggar R. A, Nagi N. M, Ali M. M, Almuasli M (2011). Quality of Life among Breast Cancer Patients in Yemen. Asian Pac J Cancer Prev.

[4] Bower J. E, Ganz P. A, Desmond K. A, Rowland J. H, Meyerowitz B. E, Belin T. R (2000). Fatigue in breast cancer survivors: occurrence, correlates, and impact on quality of life. J Clin Oncol.

[5] Cantarero-Villanueva I, Fernández-Lao C, Fernández-DE-Las-Pe-as C, Díaz-Rodríguez L, Sanchez-Cantalejo E, Arroyo-Morales M (2011). Associations among musculoskeletal impairments, depression, body image and fatigue in breast cancer survivors within the first year after treatment. Eur J Cancer Care (Engl).

[6] Coleman M. P, Quaresma M, Berrino F (2008). The CONCORD Working Group Cancer survival in five continents: a worldwide population-based study (CONCORD). Lancet Oncol.

[7] Courneya K. S, Vallance J. K, Culos-Reed S. N, McNeely M. L, Bell G. J, Mackey J. R, Friedenreich C. M (2012). The Alberta moving beyond breast cancer (AMBER) cohort study: A prospective study of physical activity and health-related fitness in breast cancer survivors. BMC Cancer.

[8] De Haes J. C. J. M, Von Knipperg E. C. E, Neijt J. P (1990). Measuring psyhological and physical distres in cancer patients: structure and application of the Rotherdam Symptom Checlist. Br J Cancer.

[9] Fayers P. M, Aaronson N. K, Bjordal K, Groenvold M, Curran D, Bottomley A (2001). On behalf of the EORTC Quality of Life Group. The EORTC QLQ-C30 Scoring Manual.

[10] Ganz P. A, Kwan L, Stanton A. L, Krupnick J. L, Rowland J. H, Meyerowitz B. E, Belin T. R (2004). Quality of life at the end of primary treatment of breast cancer: First results from the moving beyond cancer randomized trial. J Natl Cancer Inst.

[11] Gavric Z (2015). Quality of Life of Women with Breast Cancer-Emotional and Social Aspects. American Journal of Cancer Prevention.

[12] Gokgoz S, Sadikoglu G, Paksoy E, Guneytepe U, Ozcakir A, Bayram N, Bilgel N (2011). Health Related Quality of Life among Breast Cancer Patients: A Study from Turkey. Global Journal of Health Science.

[13] Goldwyn R. M (1987). Breast reconstruction after mastectomy. N England J Med.

[14] Haas B. K (2011). Fatigue, self-efficacy, physical activity, and quality of life in women with breast cancer. Cancer Nurs.

[15] Keszei A. P, Novak M, Streiner D. L (2010). Introduction to health measurement scales. J Psychosomatic Res.

[16] Monteiro-Grillo I, Marques-Vidal P, Jorge M (2005). Psychosocial effect of mastectomy versus conservative surgery in patients with early breast cancer. Clin Transl Oncol.

[17] Paterson C (2010). Quality of life measures. Br J Gen Pract.

[18] Sprangers M. A, Groenvold M, Arraras J. I, Franklin J. A, te Velde A, Muller M, Aaronson N. K (1996). The European Organization for Research and Treatment of Cancer breast cancer-specific quality-of-life questionnaire module: First results from a three-country field study. American Society of Clinical Oncology, JCO.

[19] Stanton A. L, Revenson T. A, Tennen H (2007). Health psychology: Psychological adjustment to chronic disease. Annu Rev Psychol.

[20] Ussher J. M, Perz J, Gilbert E (2012). Changes to Sexual Well-Being and Intimacy after Breast Cancer. Cancer Nurs.

[21] Victorson D, Cella D, Wagner L, Kramer L, Smith M. L, Feuerstein (2007). Measuring quality of life in cancer survivors. Handbook of cancer survivorship.

[22] WHOQOL Group (1993). Study protocol for the World Health Organization project to develop a quality of life Health Organization project to develop a quality of life assesment instrument (WHOQOL). Qual life Res.

[23] World Health Organization (2011). GLOBOCAN 2008, Section of Cancer Information.

[24] Worden J. W, Weisman AD (1977). The fallacy in post mastectomy depression. American J Med Sci.

[25] Yanez B, Thompson E. H, Stanton A. L (2011). Quality of life among Latina breast cancer patients: A systematic review of the literature. J Cancer Surviv.

[26] Young T, De Haes J. C. J. M, Curran D, Fayers P. M, Brandberg Y, On behalf of the EORTC Quality of Life Study Group (1999). Guidelines for Assessing Quality of Life in EORTC Clinical Trials.

